# Prognostic value of a novel risk classification of microvascular invasion in patients with hepatocellular carcinoma after resection

**DOI:** 10.18632/oncotarget.12547

**Published:** 2016-10-09

**Authors:** Hui Zhao, Chuang Chen, Xu Fu, Xiaopeng Yan, Wenjun Jia, Liang Mao, Huihan Jin, Yudong Qiu

**Affiliations:** ^1^ Department of Hepatopancreatobiliary Surgery, Nanjing Drum Tower Hospital Clinical College of Nanjing Medical University, Nanjing, Jiangsu, China; ^2^ Department of Hepatopancreatobiliary Surgery, Nanjing Medical University Affiliated Wuxi Second Hospital, Wuxi, Jiangsu, China; ^3^ Department of Hepatopancreatobiliary Surgery, Huai’an Hospital Affiliated to Xuzhou Medical University, Second People’s Hospital of Huai’an City, Huai’an, Jiangsu, China; ^4^ Department of Hepatopancreatobiliary Surgery, the Affiliated Drum Tower Hospital of Nanjing University Medical School, Nanjing, Jiangsu, China

**Keywords:** hepatocellular carcinoma, microvascular invasion, risk classification, prognosis

## Abstract

**Objectives:**

The present research aimed to evaluate the prognostic value of a novel risk classification of microvascular invasion (MVI) in hepatocellular carcinoma (HCC) after resection.

**Methods:**

A total of 295 consecutive HCC patients underwent hepatectomy were included in our study. We evaluated the degree of MVI according to the following three features: the number of invaded microvessels (≤5 *vs* >5), the number of invading carcinoma cells (≤ 50 *vs* >50), the distance of invasion from tumor edge (≤1 cm *vs* >1 cm).

**Results:**

All patients were divided into three groups according to the three risk factors of MVI: non-MVI group (*n*=180), low-MVI group (*n*=60) and high-MVI group (*n*=55). The overall survival (OS) and recurrence-free survival (RFS) rates of high-MVI group were significantly poorer than those of low-MVI and non-MVI groups (*P*<0.001 and *P*=0.001; *P*<0.001 and *P*=0.003). Multivariate analysis showed high-MVI, type of resection, ICG-R15 and tumor size were risk factors for OS after hepatectomy. High-MVI, type of resection and tumor size were risk factors for RFS. In subgroup analyses, the OS and RFS rates of low-MVI and non-MVI groups were better than high-MVI group regardless of tumor size. In high-MVI group, anatomical liver resection (*n*=28) showed better OS and RFS rates compared with non-anatomical liver resection (*n*=29) (*P*=0.012 and *P*=0.002).

**Conclusions:**

The novel risk classification of MVI based on histopathological features is valuable for predicting prognosis of HCC patients after hepatectomy.

## INTRODUCTION

In the world, hepatocellular carcinoma (HCC) is the sixth most common malignant tumors and the third most common cause of tumors related death [1]. With the progressing of the surgical technology, curative resection is now widely considered as the first choice of therapy for HCC [2]. Unfortunately, the high postoperative recurrence of HCC remains a serious problem. Approximately 70% of HCC patients have a recurrence within the 5 years after curative Hepatectomy [3].

The important mechanism for intrahepatic metastases is that tumor cells invade through portal vein or hepatic vein branches [4]. Microvascular invasion (MVI), defined as the invasion of tumor cells in intrahepatic portal vein or hepatic vein branches, is generally considered as a risk factor for the overall survival and recurrence rates of postoperative HCC patients [5]. Currently, MVI is only confirmed after operation by histopathological diagnosis. Previous researches reported that the prevalence of MVI ranged from 15% to 57% in HCC specimens and was associated with tumor size, alpha-fetoprotein (AFP) and typical image features [6]. Some studies indicated different histopathological characteristics of MVI had different prognostic outcomes [7, 8]. More than 50 invading tumor cells and multiple-invaded microvessels might be related to the poor survival and recurrence rates in the previous study [9]. To our knowledge, there is no definite risk classification of MVI based on histopathological characteristics.

In the present research, we retrospectively investigated clinical and histopathological characteristics of solitary HCC patients without macroscopic vascular invasion after curative hepatectomy, in order to propose a novel risk classification of MVI to predict the prognosis of HCC patients.

## RESULTS

### Patient characteristics and long-term survival

A total of 295 patients were enrolled in the present study. Overall, the median age was 55 years (range 22-87 years). Male and female patients accounted for 80% and 20%, respectively. The rates of positive HBsAg and liver cirrhosis were 80% and 67%. The mean preoperative ICG-R15 and alpha-fetoprotein levels were 5.2% (range 0.5-31.5%) and 92.6 ng/ml (range 0.7-1050000.0 ng/ml). Among histopathological characteristics, 52 (18%) and 36 patients (12%) were diagnosed with well and poorly differentiated HCC, respectively. The mean tumor size was 5.0 cm (range 1.0-12.0 cm). Anatomical resection was performed in 154 patients, and non-anatomical resection was performed in 141 patients. MVI was observed in 115 patients (40%). All patients followed up from 2 to 142 months (median 46 months). No deaths occurred in hospital. The 1-, 3-, and 5-year OS rates for all patients were 89.8%, 69.0%, and 56.6%. Correspondingly, the 1-, 3-, and 5-year RFS rates were 71.5%, 48.0%, and 38.0%.

### The relation between pathological characteristics of MVI and prognosis

Supplementary Figure 1 showed the overall and recurrence-free survival curves of different pathological characteristics of MVI in HCC patients. The group of invaded microvessels ≤ 5 (*n* = 62) significantly improved the OS and RFS rates compared with the group of invaded microvessels > 5 (*n* = 53) (HR, 0.56 [95% CI, 0.35-0.89], *P* = 0.012 and HR, 0.64 [95% CI, 0.41-0.98], *P* = 0.036). The group of invading carcinoma cells ≤ 50 (*n* = 48) showed better OS and RFS rates than the group of invading carcinoma cells > 50 (*n* = 67) (HR, 0.78 [95% CI, 0.62-0.99], *P* = 0.041 and HR, 0.63 [95% CI, 0.40-0.98], *P* = 0.036). Likewise, better OS rate was observed in the group of distance of invasion from tumor edge ≤ 1 cm (*n* = 90) compared with the group of distance of invasion > 1 cm (*n* = 25) (HR, 0.77 [95% CI, 0.59-0.99], *P* = 0.044), while there was no significant difference for the RFS rate between the two group (*P* = 0.052).

### Comparison of patient characteristics and prognosis according to the risk classification of MVI

Based on the aforementioned results, we defined three risk factors of MVI: invaded microvessels > 5, invading carcinoma cells > 50 and distance of invasion from tumor edge > 1 cm. The overall and recurrence-free survival curves of HCC patients without MVI (*n* = 180), with no risk factor (*n* = 31), one risk factor (*n* = 27), two risk factors (*n* = 47) and three risk factors (*n* = 10) of MVI was showed in Supplementary Figure 2. All HCC patients were divided into three groups according to the three risk factors of MVI: non-MVI group (*n* = 180), low-MVI group (patients with no and one risk factor, *n* = 60) and high-MVI group (patients with two and three risk factors, *n* = 55). Clinicopathological characteristics of the three groups were summarized in Table 1. There were no significant differences in age, gender, hepatitis B virus infection, background liver, Child-Pugh grade, ICG-R15, ALT, TB, AKP, Albumin, INR, Platelets, BCLC staging, type of resection, blood loss among the three groups. However, the AFP and GGT levels in non-MVI group were significantly lower than those in high-MVI group (*P =* 0.008 and *P =* 0.026). Tumor size, tumor differentiation and blood transfusion rate in non-MVI group were significantly different compared with low-MVI and high-MVI groups (*P* = 0.017 and *P* < 0.001, *P* = 0.032 and *P* = 0.012, *P* = 0.009 and *P* = 0.001).

**Table 1:** Comparative analysis of characteristics among the non-MVI, low-MVI and high-MVI groups

Variable	Non-MVI (*n* = 180)	Low-MVI (*n* = 58)	High-MVI (*n* = 57)	*P*
Age (years)^a^	54(22-81)	53(29-80)	51(24-87)	0.053
Gender				
Male	140(78)	45(78)	50(88)	0.243
Female	40(22)	13(22)	7(12)	
HBsAg				
Positive	139(77)	50(91)	47(82)	0.280
Negative	41(23)	8(9)	10(18)	
Background liver				
Noncirrhosis	68(38)	15(26)	15(26)	0.115
Cirrhosis	112(62)	43(74)	42(74)	
Child–Pugh grade				
A	176(98)	56(97)	52(91)	0.075
B	4(2)	2(3)	5(9)	
ICG-R15(%)^a^	5.0(0.5-31.5)	4.8(1.3-30.1)	5.8(2.3-26.3)	0.440
ALT(U/L)^a^	35.5(9.3-617.1)	41.3(7.5-192.0)	41.1(7.3-88.5)	0.023
TB (umol/l)^a^	15.5(5.6-47.7)	17.3(5.8-86.2)	16.3(8.4-42.6)	0.162
GGT(U/L)^a^	42.1(9.9-381.3)	52.1(15.3-683.5)	64.0(11.7-432.4) ^*^	0.026
AKP(U/L)^a^	78.5(32.8-252.6)	82.7(52.0-534.6)	86.2(53.7-216.6)	0.182
Albumin (g/l)^a^	41.9(29.3-50.8)	41.6(30.2-52.8)	41.2(23.3-49.3)	0.385
INR^a^	1.0(0.9-1.7)	1.0(0.9-1.4)	1.1(1.0-1.3)	0.185
Platelets (10^9^/l)^a^	138(4-327)	145(37-291)	161(21-331)	0.382
AFP (ng/ml) ^b^	25.3(0.7-350000.0)	130(1.3-311000.0)	258.5(2.9-1050000.0) ^*^	0.008
Tumor size(cm)^a^	4.4(1.0-12.0)	6.0(2.0-12.0) ^*^	6.0(3.0-11.0) ^*^	<0.001
BCLC staging				
0	20(11)	4(7)	1(2)	0.770
A	160(89)	54(93)	56(98)	
Tumor differentiation				
Well	42(23)	6(10) ^*^	4(7) ^*^	0.005
Moderate/Poor	138(77)	52(90)	53(93)	
Type of resection				
Anatomical	96(53)	29(50)	29(51)	0.885
Non-anatomical	84(47)	29(50)	28(49)	
Operation time (min)^a^	210(75-510)	210(90-480)	240(110-510) ^*^^#^	0.018
Blood loss (mL)^a^	300(50-2500)	400(50-3000)	500(50-2000)	0.062
Transfusion				
Yes	28(16)	18(31) *	20(35) ^*^	0.002
No	152(84)	40(69)	37(65)	

Parenthesis indicates percentage unless indicated.^a^ median (range) * *P* < 0.05 for post hoc test comparison with non-MVI ^#^ *P* < 0.05 for post hoc test comparison with low-MVI.

HBsAg, hepatitis B surface antigen; ICG-R15, indocyanine green retention rate at 15 minutes; ALT, alanine aminotransferase; TB, total bilirubin; GGT, gamma glutamyl transpeptidase; AKP, alkaline phosphatase; INR, International Normalized Ratio; AFP, alpha-fetoprotein; MVI, microvascular invasion.

The 1-, 3-, and 5-year OS rates in non-MVI group were 95.6%, 77.8%, and 69.1%, those were 91.4%, 67.0% and 49.2% in low-MVI group, and those were 70.2%, 43.3% and 27.0% in high-MVI group. In addition, the 1-, 3-, and 5-year RFS rates in non-MVI group were 79.4%, 56.6% and 47.2%, those were 74.1%, 47.8% and 32.3% in low-MVI group, and those were 43.9%, 20.6% and 15.4% in high-MVI group. Kaplan-Meier analyses revealed that OS rate in high-MVI group was obviously poorer than that in non-MVI and low-MVI groups (HR, 3.75 [95% CI, 2.51-5.59], *P* < 0.001 and HR, 2.20 [95% CI, 1.37-3.51], P = 0.001). Furthermore, poorer OS rate was observed in low-MVI group compared with non-MVI group (HR, 1.69 [95% CI, 1.09-2.62], *P =* 0.019). Similarly, the RFS rate in high-MVI group was obviously lower than that in non-MVI and low-MVI groups (HR, 2.70 [95% CI, 1.88-3.86], *P* < 0.001 and HR, 1.93 [95% CI, 1.25-2.99], *P =* 0.003). There was no significantly difference in the RFS rate between non-MVI group and low-MVI group (*P =* 0.103) (Figure 3).

**Figure 1:**
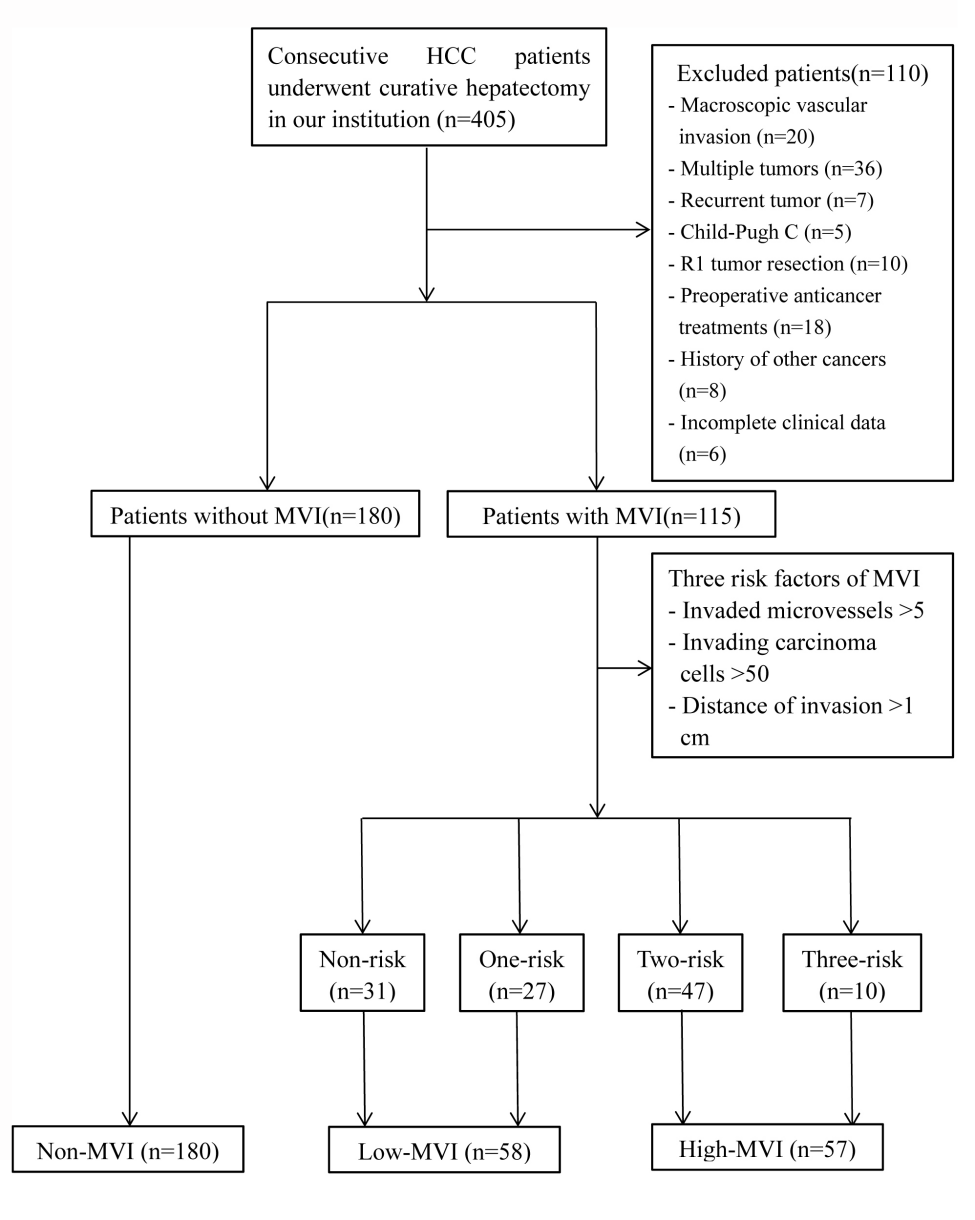
Flow diagram of enrolled patients

**Figure 2:**
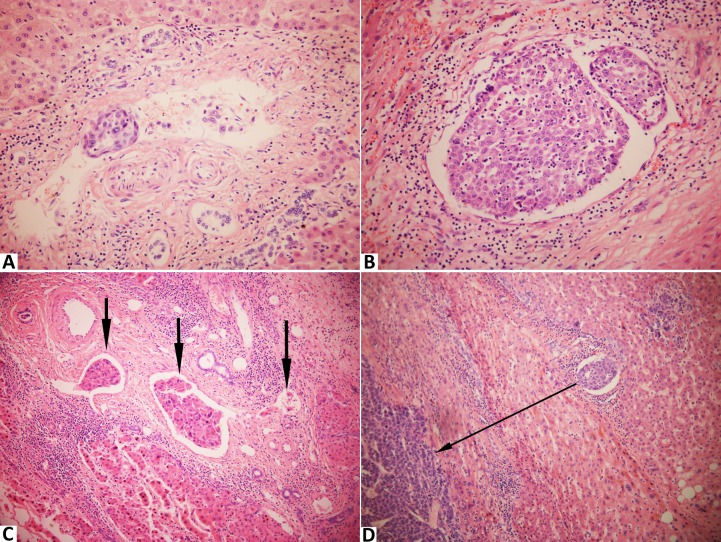
Stained with hematoxylin and eosin in hepatocellular carcinoma with microvascular invasion **A**., The number of invading carcinoma cells ≤ 50 (× 400); **B**., The number of invading carcinoma cells > 50 (× 400); **C**., The number of invaded microvessels = 3 (× 200); **D**., The distance of invasion from tumor edge = 0.3 cm (× 200).

**Figure 3:**
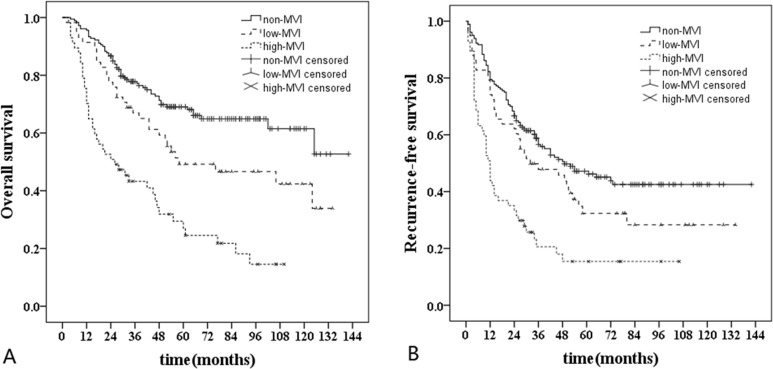
Long-term survival curves of non-MVI (*n* = 180), low-MVI (*n* = 58) and high-MVI (*n* = 57) groups **A**., Comparison of overall survival rate: high-MVI *vs* non-MVI (*P* < 0.001); high-MVI *vs* low-MVI (*P =* 0.001); low-MVI *vs* non-MVI (*P =* 0.019). **B**., Comparison of recurrence-free survival rate: high-MVI *vs* non-MVI (*P* < 0.001); high-MVI *vs* low-MVI (*P =* 0.003); low-MVI *vs* non-MVI (*P =* 0.103).

### Univariate and multivariate analyses of survival and recurrence in HCC patients after hepatectomy

Independent predictors for OS and RFS rates in HCC patients identified by univariate and multivariate analyses were illustrated in Table 2 and Table 3. Univariate analysis found that ICG-R15, Child-Pugh grade, BCLC staging, TB, INR, Albumin, tumor size, type of resection, transfusion, blood loss, tumor differentiation and high-MVI significantly influenced the OS rate. Multivariate analysis identified ICG-R15 (HR, 1.05 [95% CI, 1.00-1.09], *P* = 0.042), tumor size (HR, 1.11 [95% CI, 1.02-1.21], *P* = 0.013), anatomical liver resection (HR, 0.77 [95% CI, 0.62-0.94], *P* = 0.012) and high-MVI (HR, 2.77 [95% CI, 1.68-4.57], *P* < 0.001) as independent prognostic factors. Additionally, Child-Pugh grade, BCLC staging, Albumin, tumor size, type of resection, blood loss, tumor differentiation and high-MVI affected the RFS rate in the univariate analysis. Multivariate analysis identified tumor size (HR, 1.10 [95% CI, 1.03-1.18], *P* = 0.005), anatomical liver resection (HR, 0.79 [95% CI, 0.67-0.90], *P* = 0.002) and high-MVI (HR, 2.31 [95% CI, 1.58-3.38], *P* < 0.001) as independent prognostic factors.

**Table 2:** Univariate and multivariate analysis of prognostic factors for overall survival rate

	Univariate analysis			Multivariate analysis	
Variable	HR (95%CI)	*P*		HR (95%CI)	P
Age	0.994 (0.980-1.009)	0.442			
Gender (male)	1.030 (0.826-1.286)	0.792			
HBsAg (positive)	0.881 (0.583-1.331)	0.547			
Liver cirrhosis	0.945 (0.781-1.144)	0.564			
Child–Pugh B	3.081 (1.924-7.510)	<0.001			
ICG-R15	1.088 (1.046-1.132)	<0.001		1.046 (1.002-1.092)	0.042
BCLC A	7.445 (1.844-30.139)	0.005			
ALT	0.999 (0.995-1.003)	0.690			
TB	1.016 (1.001-1.032)	0.039			
AKP	1.001 (0.997-1.005)	0.569			
GGT	1.001 (0.999-1.003)	0.336			
Albumin	0.951 (0.913-0.989)	0.013			
INR	5.649 (1.640-19.455)	0.006			
Platelet	1.000 (0.997-1.002)	0.761			
AFP	1.000 (1.000-1.000)	0.121			
Tumor size	1.216 (1.138-1.299)	<0.001		1.113 (1.023-1.210)	0.013
Operation time	1.001 (1.000-1.003)	0.137			
Blood loss	1.000 (1.000-1.001)	0.004			
Anatomical resection	0.768 (0.646-0.914)	0.003		0.767 (0.623-0.944)	0.012
Tumor differentiation (moderate/poor)	2.427 (1.340-4.394)	0.003			
Low-MVI	1.691 (1.090-2.621)	0.019			
High-MVI	3.764 (2.527-5.697)	<0.001		2.766 (1.675-4.566)	<0.001
Transfusion (yes)	1.760 (1.211-2.558)	0.003			

HBsAg, hepatitis B surface antigen; ICG-R15, indocyanine green retention rate at 15 minutes; ALT, alanine aminotransferase; TB, total bilirubin; GGT, gamma glutamyl transpeptidase; AKP, alkaline phosphatase; INR, International Normalized Ratio; AFP, alpha-fetoprotein; MVI, microvascular invasion.

**Table 3:** Univariate and multivariate analysis of prognostic factors for recurrence-free survival rate

	Univariate analysis			Multivariate analysis	
Variable	HR (95%CI)	P		HR (95%CI)	P
Age	0.995 (0.982-1.008)	0.421			
Gender (male)	1.136 (0.778-1.659)	0.510			
HBsAg (positive)	1.025 (0.707-1.485)	0.897			
Liver cirrhosis	0.745 (0.533-1.040)	0.084			
Child–Pugh B	2.099 (1.026-3.933)	0.042			
ICG-R15	1.047 (0.997-1.097)	0.056			
BCLC A	1.916 (1.012-3.628)	0.046			
ALT	1.001 (0.999-1.004)	0.279			
TB	1.004 (0.989-1.020)	0.572			
AKP	1.002 (1.000-1.005)	0.074			
GGT	1.001 ((1.000-1.003)	0.076			
Albumin	0.960 (0.928-0.994)	0.020			
INR	2.313 (0.265-20.219)	0.448			
Platelet	1.001 (0.998-1.003)	0.577			
AFP	1.000 (1.000-1.000)	0.124			
Tumor size	1.145 (1.079-1.215)	<0.001		1.102 (1.030-1.179)	0.005
Operation time	1.001 (0.998-1.001)	0.792			
Blood loss	1.000 (1.000-1.001)	0.020			
Anatomical resection	0.776 (0.669-0.901)	0.001		0.785 (0.673-0.917)	0.002
Tumor differentiation (moderate/poor)	1.617 (1.043-2.507)	0.032			
Low-MVI	1.365 (0.935-1.991)	0.107			
High-MVI	2.715 (1.898-3.894)	<0.001		2.314 (1.583-3.382)	<0.001
Transfusion (yes)	0.864 (0.729-1.024)	0.091			

HBsAg, hepatitis B surface antigen; ICG-R15, indocyanine green retention rate at 15 minutes; ALT, alanine aminotransferase; TB, total bilirubin; GGT, gamma glutamyl transpeptidase; AKP, alkaline phosphatase; INR, International Normalized Ratio; AFP, alpha-fetoprotein; MVI, microvascular invasion.

**Figure 4:**
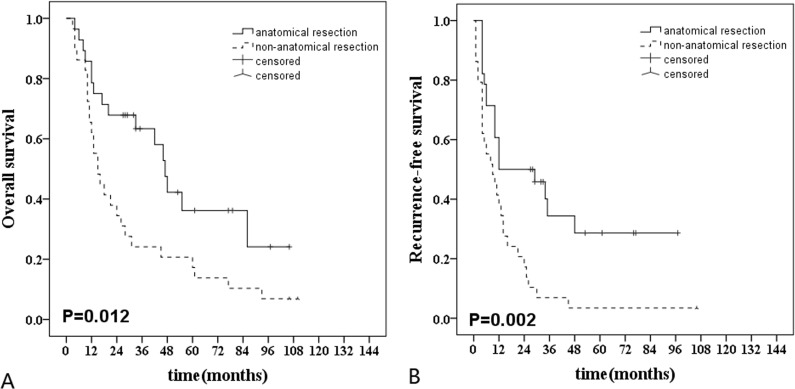
Long-term survival curves after anatomical (*n* = 28) and non-anatomic (*n* = 29) resection in hepatocellular carcinoma patients with high-MVI **A**., Comparison of overall survival rate: anatomical resection *vs* non-anatomical resection (*P* = 0.012); **B**., Comparison of recurrence-free survival rate: anatomical resection *vs* non-anatomical resection ( = 0.002).

In high-MVI group, anatomical liver resection (*n* = 28) showed better OS and RFS rates compared with non-anatomical liver resection (*n* = 29) (HR, 0.68 [95% CI, 0.50-0.93], *P* = 0.012 and HR, 0.64 [95% CI, 0.47-0.87], *P* = 0.002) (Figure 4).

### Subgroup analyses in HCC patients according to the tumor size

**Figure 5:**
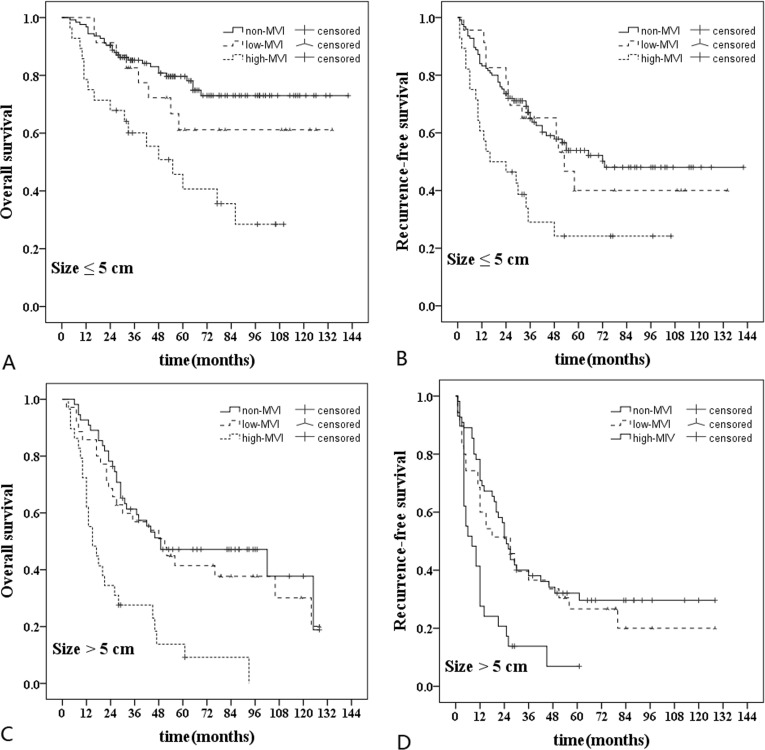
Subgroup analyses in hepatocellular carcinoma patients according to the tumor size For tumor size ≤ 5 cm (*n* = 176), **A**., Comparison of overall survival rate: high-MVI *vs* non-MVI (*P* < 0.001); high-MVI *vs* low-MVI (*P =* 0.045); low-MVI *vs* non-MVI (*P =* 0.277). **B**., Comparison of recurrence-free survival rate: high-MVI *vs* non-MVI (*P* = 0.001); high-MVI *vs* low-MVI (*P =* 0.035); low-MVI *vs* non-MVI (*P =* 0.678). For tumor size > 5 cm (*n* = 119), **C**., Comparison of overall survival rate: high-MVI *vs* non-MVI (*P* < 0.001); high-MVI *vs* low-MVI (*P* < 0.001); low-MVI *vs* non-MVI (*P =* 0.404). **D**., Comparison of recurrence-free survival rate: high-MVI *vs* non-MVI (*P* < 0.001); high-MVI *vs* low-MVI (*P =* 0.006); low-MVI *vs* non-MVI (*P =* 0.341).

Subgroup analyses were performed according to the tumor size ( ≤ 5cm *vs* > 5cm) (Figure 5). The cut-off value of tumor size determined by receiver operating characteristic curve (ROC) was 5 cm. For HCC patients with tumor size less than 5cm, the OS rate in high-MVI group (*n* = 28) was poorer than those in non-MVI group (*n* = 125) and low-MVI group (*n* = 23) (HR, 3.46 [95% CI, 1.89-6.36], *P* < 0.001 and HR, 2.30 [95% CI, 1.18-5.35], *P* = 0.045). The RFS rate in high-MVI group was poorer than those in non-MVI group and low-MVI group (HR, 2.41 [95% CI, 1.43-4.03], *P* = 0.001 and HR, 2.13 [95% CI, 1.03-4.38], *P* = 0.035). Similarly, for patients with tumor size more than 5cm, the OS rate in high-MVI group (*n* = 29) was poorer than those in non-MVI group (*n* = 55) and low-MVI group (*n* = 35) (HR, 3.40 [95% CI, 1.98-5.87], *P* < 0.001 and HR, 2.82 [95% CI, 1.56-5.12], *P* < 0.001). The RFS rate in high-MVI group was poorer than those in non-MVI group and low-MVI group (HR, 2.53 95% CI, 1.51-4.23], *P* < 0.001 and HR, 2.15 [95% CI, 1.21-3.82], *P* = 0.006). There was no significantly difference in the OS and RFS rates between non-MVI group and low-MVI group regardless of tumor size.

## DISCUSSION

In the present research, we proposed a novel risk classification of MVI on the basis of different histopathological characteristics. The OS and RFS rates of high-MVI group were significantly poorer than those of low-MVI and non-MVI groups. Although low-MVI group showed poorer OS than non-MVI group, we also noticed that tumor size in low-MVI and high-MVI groups was larger than that in non-MVI group. Multivariate analysis identified high-MVI, type of resection, ICG-R15 and tumor size were risk factors for OS after hepatectomy, while high-MVI, type of resection and tumor size were risk factors for RFS. Low-MVI was not an independent risk factor for OS and RFS. Furthermore, in order to reduce the potential bias, subgroup analyses were performed according to tumor size. Interestingly, regardless of tumor size ≤ 5 cm or > 5 cm, the OS and RFS rates of low-MVI and non-MVI groups was better than high-MVI group and no significant difference was observed between low-MVI and non-MVI groups.

MVI is the beginning of intrahepatic dissemination and metastasis in hepatocellular carcinoma [9]. Although the formation mechanism of MVI is not clear, many previous studies have identified MVI is associated with poor prognosis of HCC patients after liver resection [10, 11]. Patients with MVI are advised to perform anatomical resection and adjuvant treatments after liver resection to improve outcomes [12]. However, some other studies proposed MVI might not an independent risk factor for OS [13, 14]. Gene-expression profiling revealed that HCC with MVI was composed of two distinct phenotypes, which were less invasive and highly invasive phenotypes [15]. The highly invasive phenotype was closely related to a poor prognosis compared with the less invasive phenotype. HCC patients with different degree of MVI might result in different prognosis. But the gene microarray is difficult to be widely used in clinical practice and now MVI can be diagnosed only by histopathologic evaluation after hepatectomy. Unfortunately, there is a lack of agreement on the definition and evaluation results of MVI thus far, and the correlation between prognosis and histological feature of MVI has not been clearly identified. Furthermore, previous studies [8] revealed that different histological feature of MVI might contribute to different prognosis, so the histopathologic risk classification of MVI is valuable for predicting the prognosis of HCC patients with MVI. Because MVI is more common in HCC patients with multiple lesions and advanced tumor stage, our research only selected solitary HCC patients without macroscopic vascular invasion, which are the main candidates for curative treatments.

We defined the three histological features of MVI: the number of invaded microvessels, number of invading carcinoma cells and distance of invasion from tumor edge. The three histological features of MVI were found to be related to the OS and RFS rates of HCC patients. Ding et al. [16] suggested that the number of endothelium-coated tumor clusters was associated with poor prognosis and micrometastasis of HCC after hepatectomy, and endothelium-coated tumor clusters was defined MVI now. Roayaie et al. [17] found the distance of microvascular invasion > 1cm was an independent risk factor for OS rate of HCC patients. Based on the three risk factors of MVI, we divided all the patients into the non-MVI, low-MVI and high-MVI groups. Significant difference in prognosis among the three groups was observed. Sumie et al. [7] proposed a risk classification according to the number of invaded microvessels in study of 207 HCC patients meet the Milan criteria. They divided the patients into the severe MVI (number of invaded microvessels > 5) and mild MVI groups (number of invaded microvessels ≤ 5). In survival analysis, significantly difference was observed in OS rate, but not in RFS rate between the two groups. Compared with our research, there were some significant differences. First, only one histological feature of MVI was taken into account in Sumie’s research. The number of invading carcinoma cells and distance of invasion from tumor edge were reported to be associated with the prognosis of HCC patients. Second, the patients with hepatitis C virus (HCV) infection comprised 76% of the total patients in Sumie’s research. However, in our cohort, nearly 80% was patients with hepatitis B virus (HBV) infection and no HCV patients were included. In another study about the risk classification of MVI, Fujita et al. [18] indicated that the number of invaded microvessels ( ≥ 2) and invading carcinoma cells ( > 50) resulted in poorer prognosis. However, the definition of MVI in the study was only confined to the portal vein and did not included hepatic vein invasion, which was different from the present definition of MVI.

Many researches about predicting MVI of HCC before treatment were performed in recent years. High level of AFP was found to be associated with MVI [19, 20]. In the present study, the level of AFP in high-MVI group was significantly higher than that in low-MVI and non-MVI groups. There was no significantly difference between low-MVI and non-MVI groups. You et al. [19] analyzed 215 patients who underwent liver resection, and showed the AFP level greater than 400ng/mL was independently associated with MVI. Jin et al. [21] elaborated that the high AFP mRNA level of circulating tumor cells could be a valuable predictor for HCC metastasis after liver resection. Circulating tumor cells might be an important formation mechanism of MVI [22]. These results could explain the relevance between the AFP level and high-MVI. Therefore, the AFP level might be an independent predictor of MVI, especially high-MVI. Larger tumor size and poorer tumor differentiation were observed in high-MVI groups compared with non-MVI group in our study, which was consistent with the previous studies [23, 24]. But it is difficult to identify tumor differentiation before surgery due to heterogeneity in the solitary tumor. Tumor size more than 5 cm was reported to be strongly related to the prevalence of MVI [25]. However, Yamashita et al. [26] found there was no significant correlation between tumor size and MVI, and 43 patients (28.9%) were found MVI in 149 patients with HCC ≤ 2 cm. Furthermore, a 35-gene signature was identified to be associated with the presence of vascular invasion, but the study was mainly based on Caucasian patients and the accuracy was only 69% [27]. Generally, many preoperative factors, such as tumor size, serum biomarkers and gene signature of HCC, have been found to be closely associated with MVI. However, the sensitivity and specificity of each predictive factors are not high and these results need further validation in the clinical research. Our previous studies [28, 29] explored the significance of gross classification on solitary HCC after liver resection. The invasive growth type of HCC was closely related with the incidence of high-MVI. Therefore, the gross classification on solitary HCC, which is a great predictor of MVI, may provide a basis for surgical procedure selection.

Anatomical liver resection was an independent prognosis risk factor for the OS and RFS rates in the present research. Especially in high-MVI group, anatomical liver resection significantly improved the OS and RFS rates compared with non- anatomical liver resection. HCC has a high tendency to invade the intrahepatic vascular system and spreads through the branch, which is the main route for the formation of MVI [4]. Shi et al. [30] revealed that 92% of all MVI could extend through intrahepatic vascular system in the 2 cm range of distance from the main tumor. Ueno et al. [31] also found the distance of micrometastases from the main tumor was 9.5±6.2 mm in the non-boundary type of HCC. In our study, patients with MVI (distance of invasion > 1 cm) account for 22%, and the furthest distance was 2 cm. Anatomical liver resection could completely remove the tumor-bearing portal tributaries in order to eliminate macroscopic and microscopic metastases in the liver. A multicenter study by Italian and Chinese researchers showed anatomical liver resection significantly improved the RFS rate in aggressive HCC (MVI or poor tumor differentiation) [32], which supported our results. Additionally, for HCC patients with high-MVI, adjuvant treatment strategies after operation might be considered, such as postoperative adjuvant transcatheter arterial chemoembolization (TACE). A meta-analysis suggested TACE could improve OS rate in HCC patients with macroscopic vascular invasion [33]. Although Sun et al. [34] analyzed 322 HCC patients with MVI and showed postoperative adjuvant TACE to be an independent risk factor for RFS and OS, the prognosis of HCC patients with MVI for postoperative adjuvant TACE is still controversial. We believe that definite risk classification of MVI can contribute to further investigate the effectiveness of postoperative adjuvant TACE on HCC patients with MVI.

There were some limitations in the present study. First, it was a single-center research, and the risk classification of MVI based on different histopathological characteristics needs to be validated in other center. Second, a prospective randomized control trial is required to further confirm the treatment program for HCC patients with high-MVI. To the best of our knowledge, a randomized control trial about the comparison between sorafenib and TACE for MVI in HCC patients after radical resection is performing now (registered on ClinicalTrials.gov, NCT02537158). Finally, because the classification is based on histopathologic evaluation after hepatectomy, specific serum markers and genes are needed to predict high-MVI before treatment.

In conclusion, our study indicated the risk classification of MVI based on the number of invaded microvessels, number of invading carcinoma cells and distance of invasion from tumor edge is valuable for predicting prognosis of HCC patients without macroscopic vascular invasion after curative hepatectomy.

## PATIENTS AND METHODS

### Study population

From January 2004 to December 2013, a total of 405 consecutive HCC patients underwent curative hepatectomy in our institution. To clearly evaluate the real prognostic impact of MVI, 110 patients were excluded for the following reasons: (1) macroscopic vascular invasion (*n* = 20), (2) multiple tumors (*n* = 36), (3) recurrent tumor (*n* = 7), (4) Child-Pugh C (*n* = 5), (5) R1 tumor resection (*n* = 10), (6) presence of any preoperative anticancer treatments (*n* = 18), (7) a history of other cancers (*n* = 8), (8) incomplete clinical data (*n* = 6) (Figure 1). The present study was carried out in accordance with the Declaration of Helsinki revised in 1983. The retrospective study was approved and exempted from the requirement to obtain informed consent by the Committee on Medical Ethics of Nanjing Drum Tower Hospital.

### Clinical characteristics

Preoperative laboratory examinations and operation information were retrospectively reviewed. Age, gender, hepatitis B surface antigen (HBsAg), serum alanine aminotransferase (ALT), glutamyl-transpeptidase (GGT), alkaline phosphatase (AKP), serum total bilirubin (TB), serum albumin (ALB), alpha-fetoprotein (AFP), platelet count (PLT), international normalized ratio (INR), Child-Pugh grade, background liver, indocyanine green retention rate at 15 minutes (ICG-R15), BCLC staging, tumor size, operation time, blood loss, and blood transfusions. Anatomical resection was charactered as any type of complete excision at least one segment based on Couinaud’s classification [35], included segmentectomy, subsegmentectomy, sectoriectomy and hemihepatectomy. Non-anatomical resection was defined as limited resection or enucleation without regard to the Couinaud’s segmental, sectoral structure. The indications for the hepatectomy and the type of operation were usually based on the tumour location, remnant liver volume and the hepatic functional reserve assessed by ICG-R15 and Child-Pugh grade.

### Histopathological characteristics

All the resected specimens were cut into approximately 3 to 5 mm thick slices and fixed in 1 % formalin for further pathological examination. The liver slices, which contained tumor tissues and non-cancerous adjacent normal tissues, were embedded in paraffin, cut into 4-μm sections, and stained with hematoxylin and eosin. At least a slice of normal liver parenchyma 1 cm away from the tumor edge was examined. The extent of tumor differentiation was evaluated as well, moderate and poor according to Edmondson-Steiner grading system [36]. MVI was defined as the invasion of tumor cells in a portal vein, hepatic vein, or a large capsular vessel of the surrounding hepatic tissue, partially or totally lined by endothelial cells that was visible only on microscopy [37]. We evaluated the degree of MVI according to the following three features based on all the sections of each case: the number of invaded microvessels ( ≤ 5 *vs* > 5), the number of invading carcinoma cells ( ≤ 50 *vs* > 50), the distance of invasion from tumor edge ( ≤ 1 cm *vs* > 1 cm) (Figure 2). All the histopathological evaluations were performed by two independent pathologists (C. J. and S. J.) blinded to the clinical characteristics.

### Patient Follow-up

After discharge, all patients were followed up regularly by the serum levels of AFP, liver function and abdominal ultrasonography every month in the first half a year, then every three months in the next one and a half years and every half a year in the later time. Contrast-enhanced computed tomography (CT) was performed every 4 months. Recurrence should be confirmed by at least two imaging modalities, such as CT and enhanced magnetic resonance imaging (MRI). After the detection of a recurrence, further treatment such as repeat hepatectomy, local ablation, transcatheter arterial chemoembolization (TACE), or other therapeutic modalities, including molecular targeted therapy would be undertaken. Overall survival (OS) was defined as the time interval between the operation and the date of the death. Recurrence-free survival (RFS) was defined as the period after the operation when a recurrence could be detected. Follow-up data were collected until December 31, 2015.

### Statistical analysis

Categorical data were compared by the chi-square test and continuous variables were compared using the Kruskal-Wallis test with Bonferroni correction followed by a post hoc test. The OS and RFS rates groups were calculated according to the Kaplan-Meier survival curves and compared by the log-rank test. Prognostic risk factors were analyzed by using univariate and multivariate Cox proportional hazards models. Clinical characteristics were statistically significant in univariate analysis were subsequently included in a multivariate analysis. For all tests, *P* values < 0.05 were considered statistically significant. Statistical analysis was performed using SPSS version 21.0 (SPSS Inc., Chicago, IL).

## SUPPLEMENTARY FIGURES


