# Supplementary material for: Prognostic value of a novel risk classification of microvascular invasion in patients with hepatocellular carcinoma after resection
Source: Oncotarget. 2016 Oct 9;8(3):5474–86. doi: 10.18632/oncotarget.12547 (PMC5354924; doi:10.18632/oncotarget.12547)
Supplement: Supplementary file 1 [file oncotarget-08-5474-s001.pdf]

Prognostic value of a novel risk classification of microvascular invasion in patients with hepatocellular carcinoma after resection

Supplementary Material

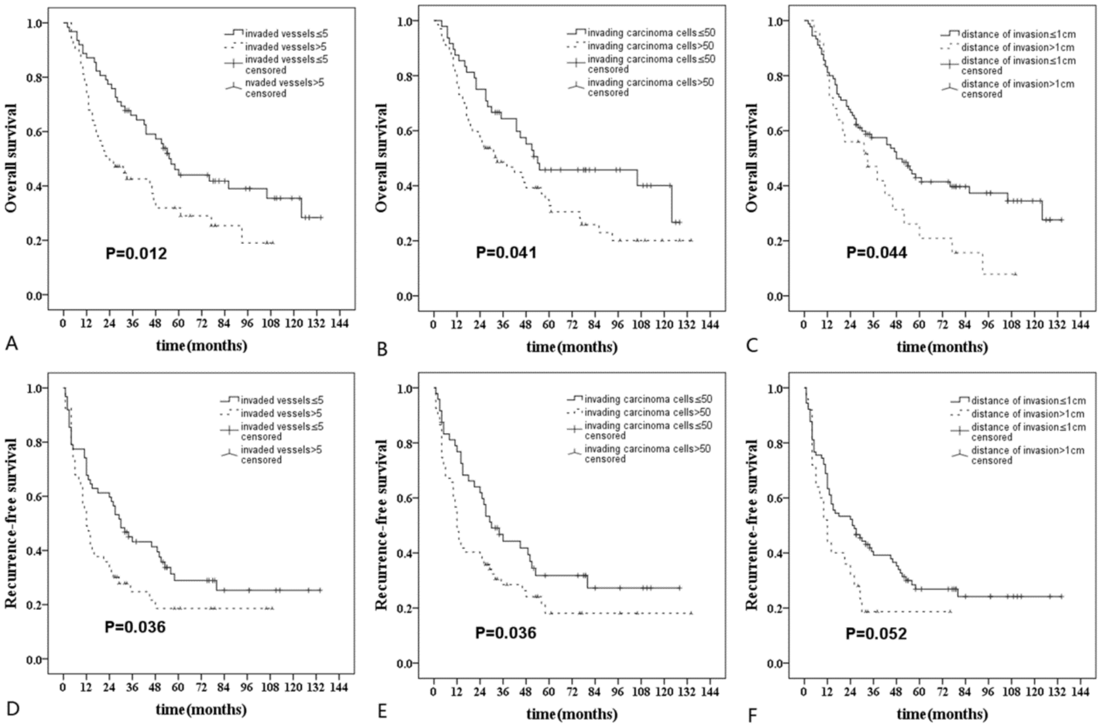

Supplementary Figure 1: The overall and recurrence-free survival curves of different pathological characteristics of microvascular invasion in hepatocellular carcinoma patients. **A**, Comparison of OS rate (*P*=0.012) and **D**, RFS rate (*P*=0.036) in the group of invaded microvessels ≤ 5 (n=62) and > 5 (n=53); **B**, Comparison of OS rate (*P*=0.041) and **E**, RFS rate (*P*=0.036) in the group of invading carcinoma cells ≤ 50 (n=48) and > 50 (n=67); **C**, Comparison of OS rate (*P*=0.044) and **F**, RFS rate (*P*=0.052) in the group of distance of invasion from tumor edge ≤ 1 cm (n=90) and > 1 cm (n=25).

OS, overall survival; RFS, recurrence-free survival.

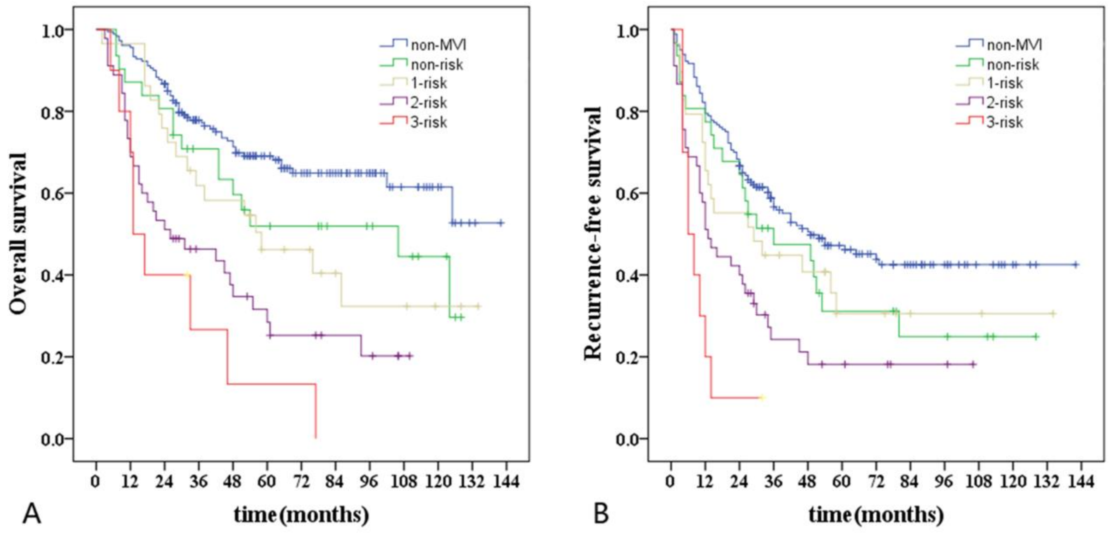

Supplementary Figure 2: A, The overall (*P* <0.001) and **B**, recurrence-free survival (*P* <0.001) curves of hepatocellular carcinoma patients without microvascular invasion (n=180), with no risk factor (n=31), one risk factor (n=27), two risk factors (n=47) and three risk factors (n=10) of microvascular invasion.
